# Think-Aloud Modelling for Clinical Reasoning in Undergraduate Dental Education: A Pilot Study

**DOI:** 10.3390/dj14100634

**Published:** 2026-10-02

**Authors:** Rohit Kunnath Menon, Tasnim Ahmad Ghanem, Haya Mohamad Hajjo

**Affiliations:** 1 Department of Clinical Sciences, College of Dentistry, Ajman University, Ajman, PO: 346, United Arab Emirates; 2 College of Dentistry, Ajman University, Ajman, PO: 346, United Arab Emirates

**Keywords:** clinical reasoning, decision making, problem solving, treatment planning, dental education

## Abstract

**Background:** The purpose of the study was to explore the impact of a think-aloud (TA) pedagogical approach on the enhancement of clinical reasoning skills through the use of think-aloud recordings. **Methods:** Fifth-year dentistry students who provided written informed consent for participation were included in this study. During phase 1 (TA1), the expert recorded five think-aloud videos verbalizing his thoughts to analyze the clinical data and devise treatment options. Ten students completed a knowledge assessment test and recorded their own think-aloud recordings for each case, resulting in 50 verbal transcripts. Subsequently, they accessed think-aloud recordings of the expert through Moodle. During the second phase (TA2), an identical protocol was implemented for both students and the expert with five new cases, resulting in the production of 55 additional verbal transcripts. The agreement of student and expert thoughts as recorded by the transcripts was evaluated through quantitative analysis, which employed Cohen’s kappa and Wilcoxon signed rank statistical tests. Qualitative thematic analysis was employed to investigate the convergence of thinking tendencies over time. R version 4.5.2 was used for the statistical analysis and for generating the word clouds. **Results:** The percentage of agreement between students and the expert increased for pre-treatment considerations [43.07% (TA1) to 79.4% (TA2)] and perceived challenges [60.76% (TA1) to 62.94% (TA2)], whereas it declined for treatment options [63.07% (TA1) to 44.70% (TA2)]. The Cohen’s kappa values for agreement did not exhibit a statistically significant increase (V = 13, *p* = 0.16). The number of major congruent themes between the students and the expert doubled following exposure to the expert recordings. The number of shared terms in the transcripts increased from 65 (TA1) to 90 (TA2). **Conclusions:** This exploratory investigation identified different patterns of student–expert agreement. Agreement increased for pre-treatment considerations, remained relatively stable for anticipated challenges, and decreased for treatment options. Longitudinal studies with a larger sample size are recommended to study these trends. Think-aloud recordings warrant further investigation as a potential educational resource to enhance clinical reasoning.

## 1. Introduction

Treatment planning can be paralleled with a decision-making process where cognitive processes intertwine before arriving at a conclusion or lead to further investigations. The end results of the process seldom provide a glimpse of the inherent cognitive process that culminated in the final output. Competency in clinical reasoning and decision-making underscores a key clinical area which encompasses affective and cognitive domains of a learner [1].

Deductive diagnosis primarily depends on non-analytical linking of patterns saved in the memory of a learner which in turn leads to deduction of a definitive hypothesis by confirming or refuting decisions based on additional data. In most cases, a combination of pattern recognition and hypothetical deductions may be employed [2]. Clinical reasoning skills have been shown previously to vary considerably depending on the level of experience of a clinician [3]. A novice primarily establishes a hypothetical diagnosis first and tries to establish this by additional data, wherein an experienced clinician may deploy a forward reasoning strategy to gather data and then establish the diagnosis. Psychometric measurements have pointed to the personalized nature of the clinical reasoning process and established the emphasis of individual thought processes [4]. The development of the clinical reasoning process follows a multi-trajectorial framework traversing through pre-structural, unistructural, multi-structural and relational frameworks finally leading to advanced reasoning [5,6]. Establishing these competency layers with minimal errors may take years of clinical experience and exposure to a diverse array of clinical cases and scenarios. Traditionally, case-based learning [7], problem-based learning [8], small-group learning [9], and lectures have been integrated into the teaching curricula to train and support students in developing their critical thinking abilities for treatment planning. A recent systematic review has established the replacement of these conventional methods by virtual patients, AI-based modules, SNAPPS, mobile apps, and integrated curricula [10]. However, the limited clinical experience of students presents a substantial barrier for developing skills in the cognitive domain owing to the rudimentary structural blocks that are embedded in the minds of a novice learner. A peek into the mind of an expert or teacher may be invaluable for early-stage learners who are in the formative process of developing a cognitive pattern for clinical reasoning.

A think-aloud interview is an established educational approach to capture the thought process of an individual with minimal intervention from the interviewer [11]. The think-aloud protocol involves the collection of verbal data in a concurrent or retrospective format followed by analysis of the verbal transcripts [12]. The verbal transcripts provide insight into the cognitive process of an expert which may play a vital role in establishing evidence-based thinking and development of novel cognitive patterns in novice learners. Even though think-aloud approaches have been previously used in pharmacy [13,14] and nursing [15,16], employment in undergraduate courses in medicine [17] and dentistry [18] has been relatively scant. Previous studies have reported favourable student feedback on using think-aloud recordings to teach pre-clinical skills [19] and clinical reasoning skills in prosthodontics [20] in the undergraduate dental curricula. This study aimed to explore and evaluate whether exposure to the thought process of an expert through a think-aloud learning resource will have an impact and modify the thought process of undergraduate dental students.

## 2. Materials and Methods

Ethical approval for the study was obtained from the research ethics committee of Ajman University before commencement of the study and recruitment of study participants (Project Number: D-F-H-24-10-31).

Participants and equity in research: A total of 150 fifth-year undergraduate dental students were invited to participate in the study. An email invitation was sent to the students with the study information sheets and informed consent sheet attached. If the students requested additional information, a meeting was arranged to explain the study flow to the students. Participation was voluntary and students were assured that participation or declining to participate would not affect any aspects of teaching and grading for the course. The learning resource that was created was made available to all students in the course after completion of the research activities irrespective of their participation status.

**Figure 1:**
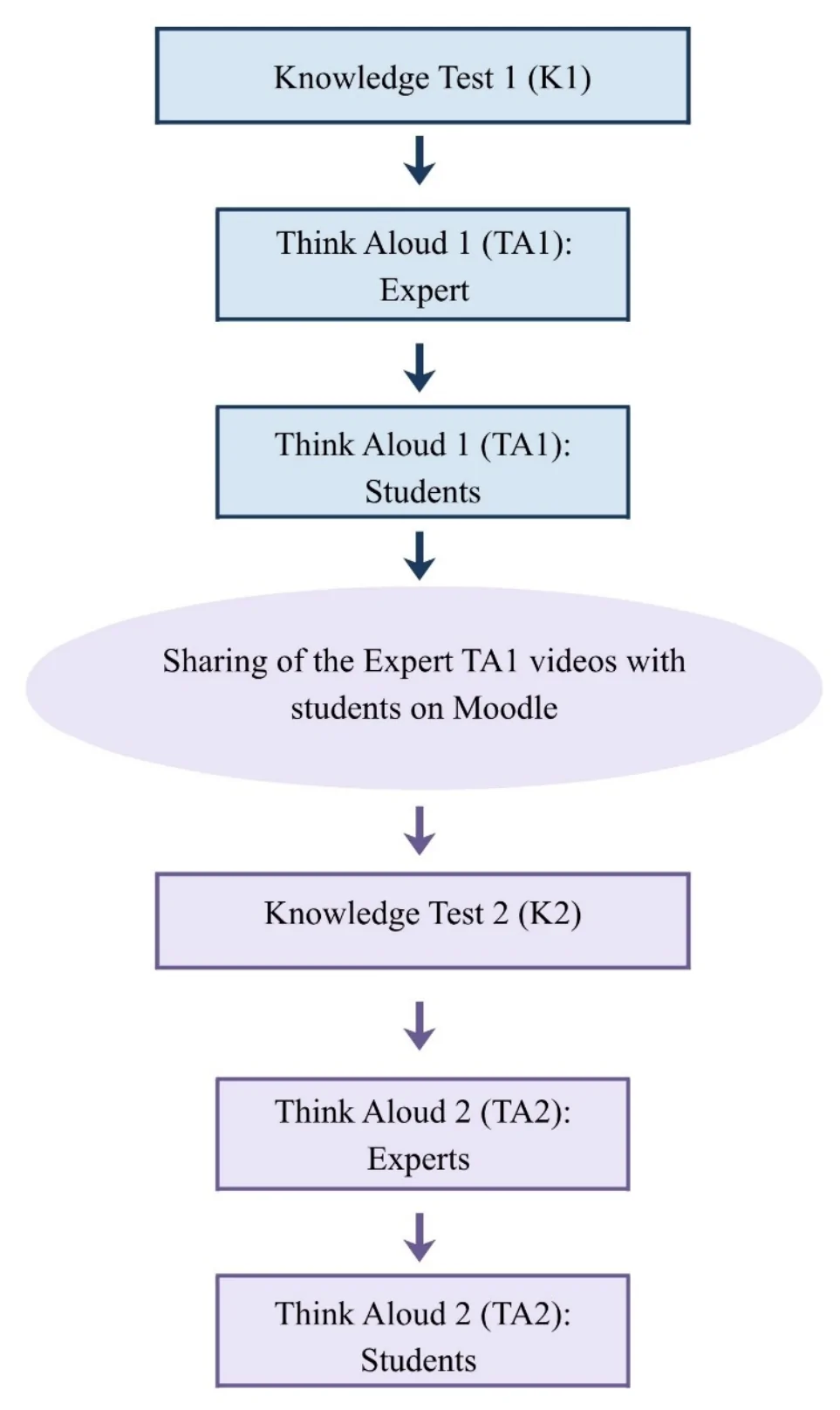
Study Flowchart. TA: Think-aloud, K: Knowledge.

Preparation of the content and vetting: The specialist prosthodontist (expert) who was the primary instructor for the course compiled clinical information (clinical data, radiographic images, and data from investigations) for 10 clinical cases in prosthodontics. The selected cases were the same cases treated by the cohort in their clinical sessions. These cases were embedded into PowerPoint slides. The diagnosis was indicated and the treatment plan was listed correctly (Emergency treatment followed by control of active diseases and definitive restorations/prostheses). The diagnosis and treatment planning list were used as the ground truth for analysis of student responses and were not visible to the students. The content underwent vetting from a group of three specialists to ensure accuracy and evidence-based content. Five cases were used for the first think-aloud session and the remaining five were used for the second think-aloud session. The overall content and learning outcome of the cases in the first and second think-aloud sessions were the same, namely “Treatment options for replacement of missing teeth.” The study flow is depicted in Figure 1.

Knowledge Test 1 (K1): Ten multiple-choice questions were prepared by the specialist primary instructor, based on the learning outcomes from the selected cases. The content underwent vetting from a group of three specialists to ensure accuracy and evidence-based content. A knowledge test was conducted for the 10 students who volunteered to be part of the study. One mark was awarded for each correct answer.

Think-aloud 1 (TA1): The five prepared cases were used by the expert (primary instructor of the course) to record five think-aloud videos (mp4 files) in which the expert verbalized and recorded his thoughts to analyze the clinical data and then devise the treatment options. A think-aloud learning resource was created in the MOODLE learning platform containing the video/audio recordings (mp4 files) and was stored as “hidden from student view”. The recorded files were saved and transcribed.

Think-aloud 1 (TA1) (Students): All 10 participating students were “trained” to verbalize their thoughts by providing simple mathematical calculations and dummy cases. Each student recorded 5 think-aloud sessions where they verbalized their thoughts to analyze the clinical data and devise the treatment options. The facilitator did not provide feedback or assist the student during the session. No time limit was imposed. The recorded files were saved and transcribed.

Sharing of the TA1 videos with students: Subsequently, the expert think-aloud learning resource was shared with the students, and the students were given two weeks to use the resource. The students listened to the recordings of the expert and compared their treatment options and thought process with those of the expert. To ensure fairness, the expert recordings were shared with all students in the cohort for learning purposes.

Knowledge Test 2 (K2): A second knowledge test was conducted for the 10 students. Ten multiple-choice questions were prepared by the expert primary instructor, based on the same overarching learning outcome as for the previous test, from the selected cases. One mark was awarded for each correct answer.

Think-aloud 2 (TA2): The five new prepared cases were used by the expert to record five new “Think-aloud” videos (mp4 files). The new cases went through the same standardization vetting protocol as for the first batch. Further, the learning outcome and case complexity were similar to those of the first set of videos. The recorded files were saved and transcribed.

Think-aloud 2 (TA2) (Students): The 10 students generated think-aloud recordings for the five new cases. The recorded files were saved and transcribed.

Quantitative Assessment: R version 4.5.2 was used for all statistical analyses. Verbal transcripts were coded as per the three pre-defined domains: pre-treatment considerations (PT), treatment options (TO), and anticipated challenges (C). Dichotomous coding as 1 or 0 was undertaken for identification of individual clinical reasoning elements in the verbal transcripts. The agreement with the expert was assessed for each student at the level of every coded element. Uniform student and expert codes (1/1 or 0/0) represented concordance, whereas divergent codes (1/0 or 0/1) indicated conflict. To get the singular Cohen’s kappa (κ) value for each student at each timepoint, the binary observations from the three domains (PT, TO, and C) were amalgamated into a unified vector for that student and juxtaposed with the corresponding expert vector. Consequently, the reported κ signifies the overall student–expert agreement across the three domains, rather than an average of the κ coefficients relevant to each area. The percentage of agreement was determined as the ratio of matching classifications between students and the expert. Given the non-parametric character of the data, the Wilcoxon signed-rank test was used to compare the κ values obtained at Timepoint 1 (TA1) and Timepoint 2 (TA2) in order to assess changes in agreement over time. The threshold for statistical significance was fixed at *p* < 0.05. To offer a descriptive measure of concordance, percent agreement between student and expert responses was computed for each case in addition to κ statistics.

Qualitative Assessment: The qualitative information obtained from transcript recordings was examined using thematic analysis. Using an inductive approach, transcripts from both students and the expert at TA1 and TA2 were systematically coded and grouped into overarching themes, enabling themes to emerge directly from the data without a priori assumptions. The transcripts were independently coded by TAG and HMH. Any disagreements regarding code or theme allocation were resolved through discussion with RKM. Themes were analysed both within and between timepoints for both groups to investigate patterns of similarity and difference. Thematic congruence was defined as the presence of conceptually corresponding themes in the student and expert transcripts, even when the specific terminology used differed. The number of congruent themes was compared between TA1 and TA2 to examine changes in the alignment of student-derived themes with expert themes. Additionally, following text preparation, including the removal of stop words, common terms between student and expert transcripts were identified to quantify lexical overlap. Word clouds were also created to graphically depict the most frequently occurring terms in each transcript.

## 3. Results

**Figure 2:**
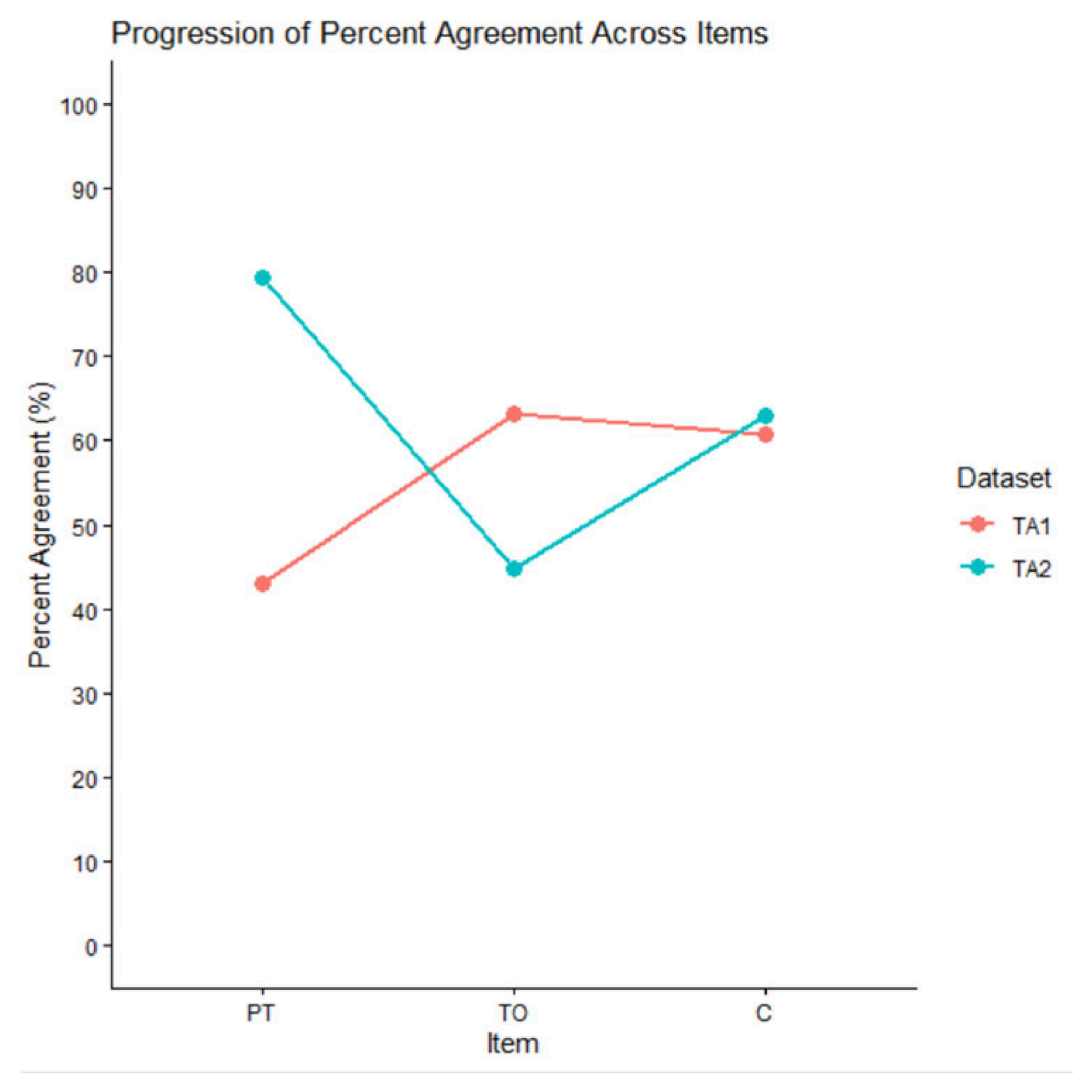
Progression plot per item. TA: Think-aloud, PT: Pre-treatment considerations, TO: Treatment options, C: Challenges.

Quantitative: Knowledge scores did not differ significantly between the pre-assessment (6.60 ± 1.3) and post-assessment (5.80 ± 1.6) (paired-samples *t*-test, t (9) = 1.35, *p* = 0.210). Percent agreement between students and the expert for pre-treatment considerations [43.07% (TA1), 79.4% (TA2)], treatment options [63.07% (TA1), 44.70% (TA2)] and perceived challenges [60.76% (TA1), 62.94% (TA2)] is depicted in Figure 2.

Cohen’s Kappa values for agreement in pre-treatment considerations, treatment options and challenges in each case varied from 0.15 to 0.42 (TA1) and from 0.27 to 0.42 (TA2) (Table 1).

**Figure 3:**
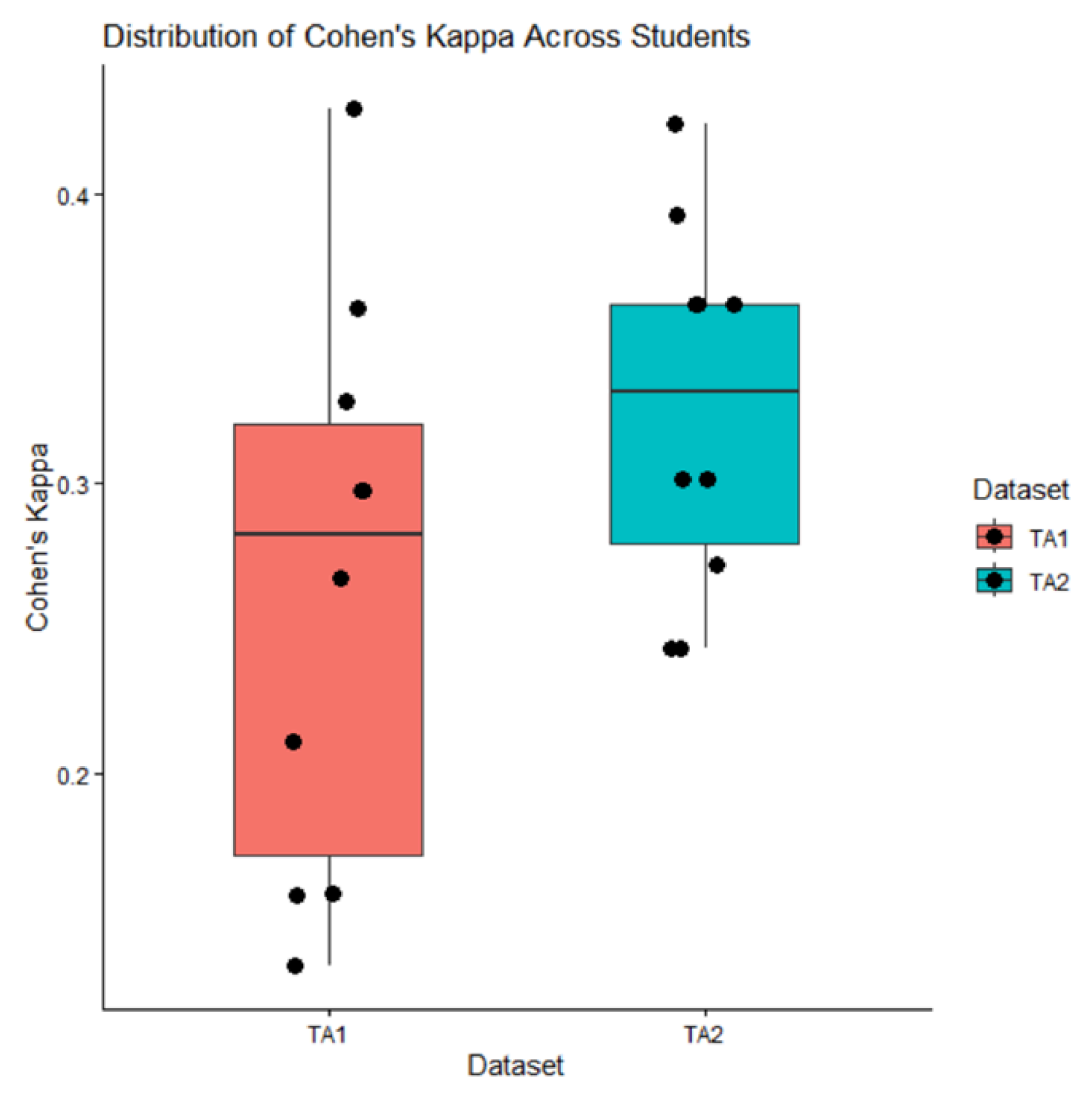
Boxplot comparing the Cohen’s kappa values between students and the expert. TA: Think-aloud.

The Wilcoxon signed rank exact test (V = 13, *p* = 0.16) revealed that the difference in agreement was not statistically significant (Figure 3).

**Figure 4:**
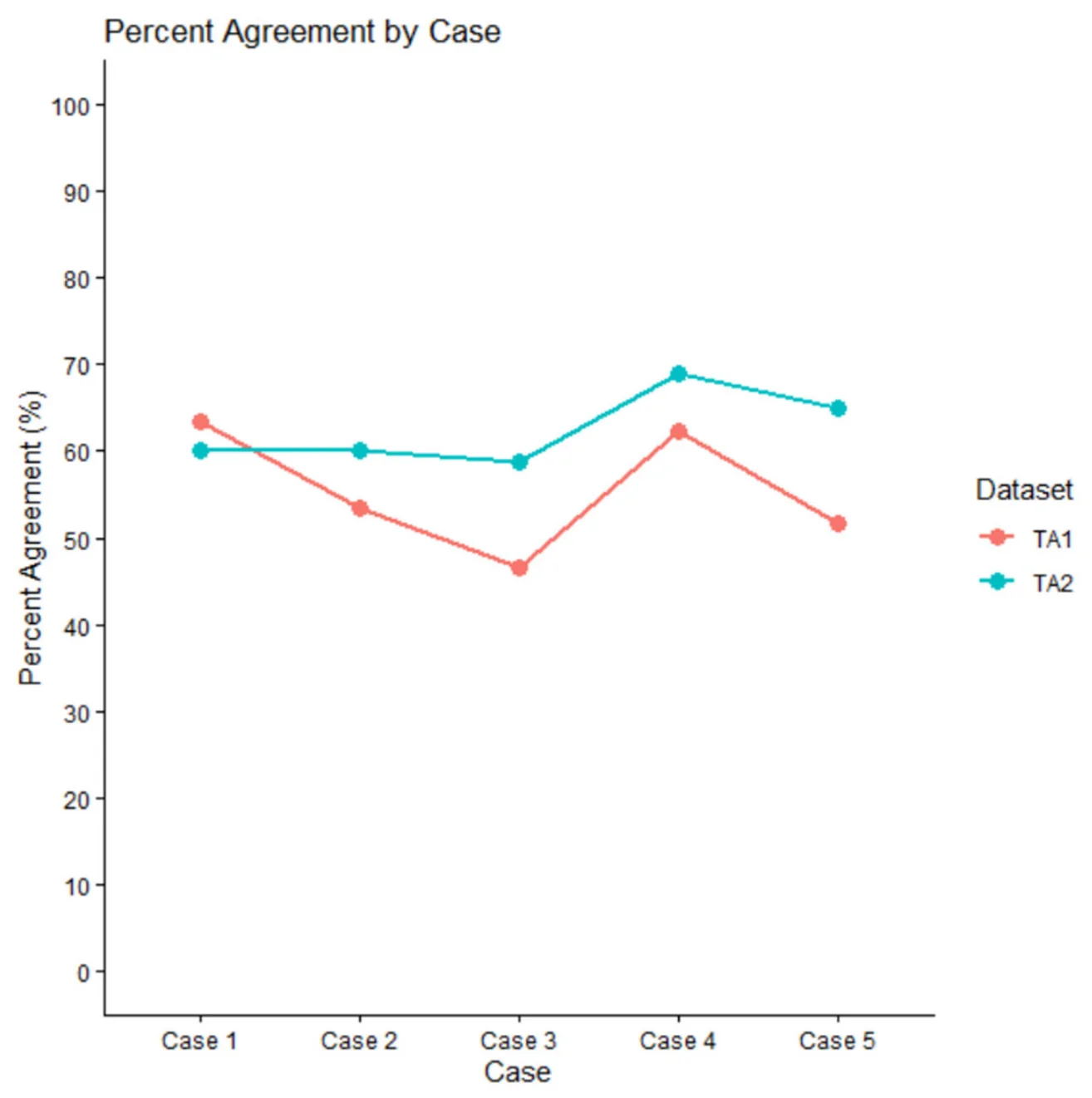
Progression plot per case. TA: Think-aloud.

Percent agreement for cases ranged from 51% to 63% (TA1) and from 58% to 65% (TA2) (Figure 4).

Qualitative: For TA1, six themes were identified from the transcripts of the verbal recording by the expert as compared to seven themes for students. The themes and representative comments for the expert and students are depicted in Table 2.

**Figure 5:**
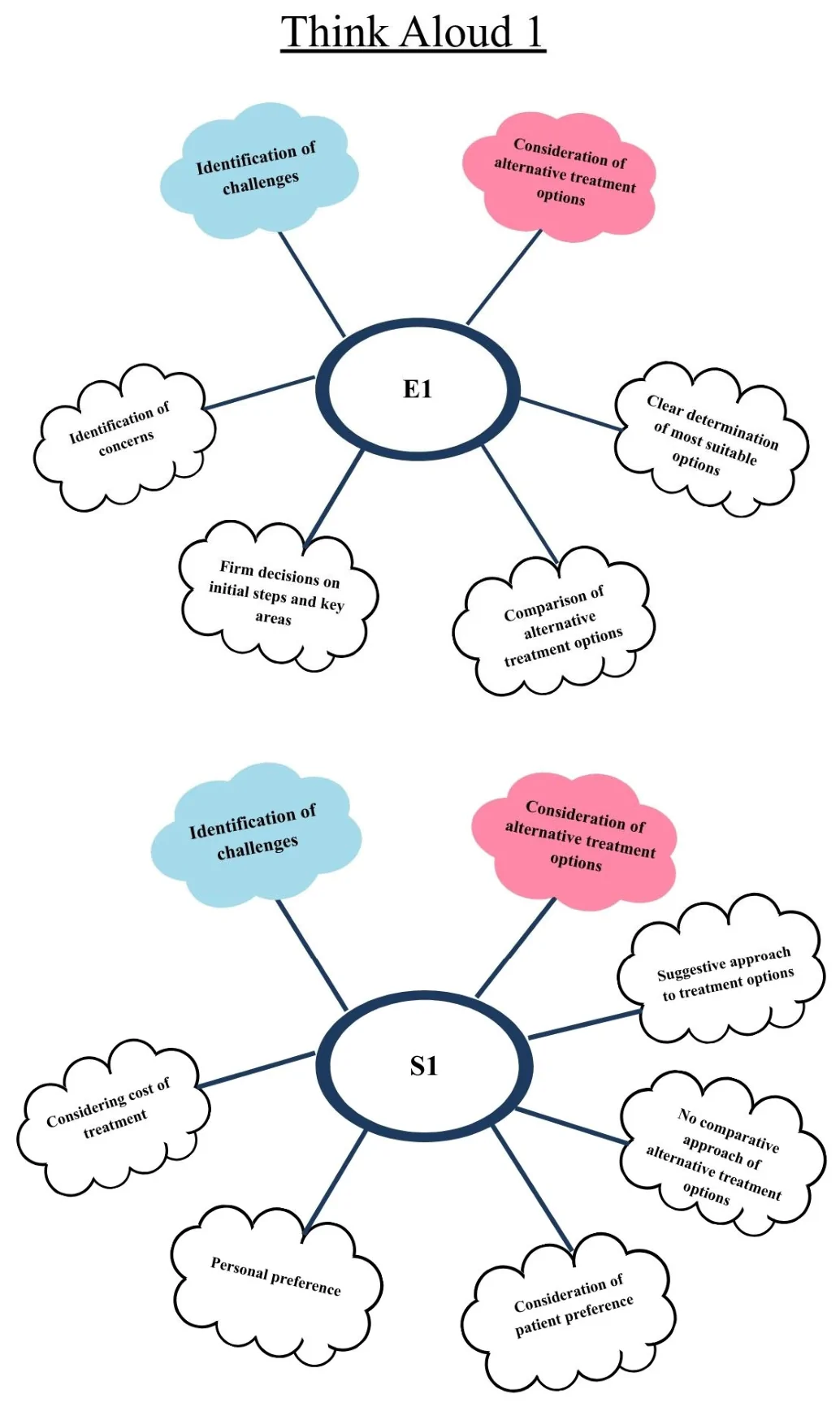
Thematic congruence mindcloud (TA1). TA: Think-aloud, E: Expert, S: Student. Same themes are indicated in the same colour.

Two congruent themes were identified between the expert and students (Figure 5).

For TA2, six themes were identified from the transcripts of the verbal recording by the expert as compared to five themes for students. The themes and representative comments for the expert and students are depicted in Table 3.

**Figure 6:**
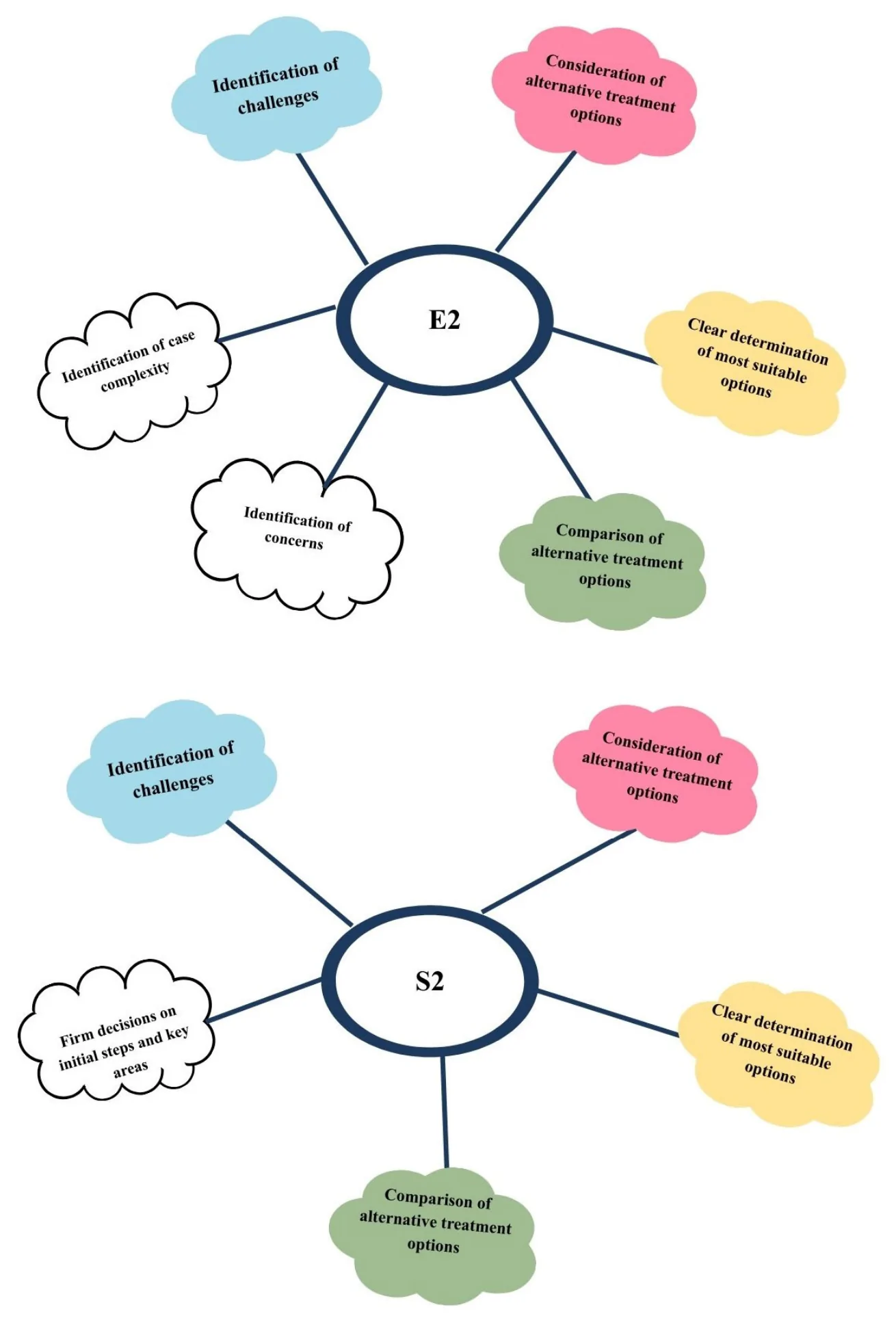
Thematic congruence mind cloud (TA2). TA: Think-aloud, E: Expert, S: Student. Same themes are indicated in the same colour.

Four congruent themes were identified between the expert and students (Figure 6).

**Figure 7:**
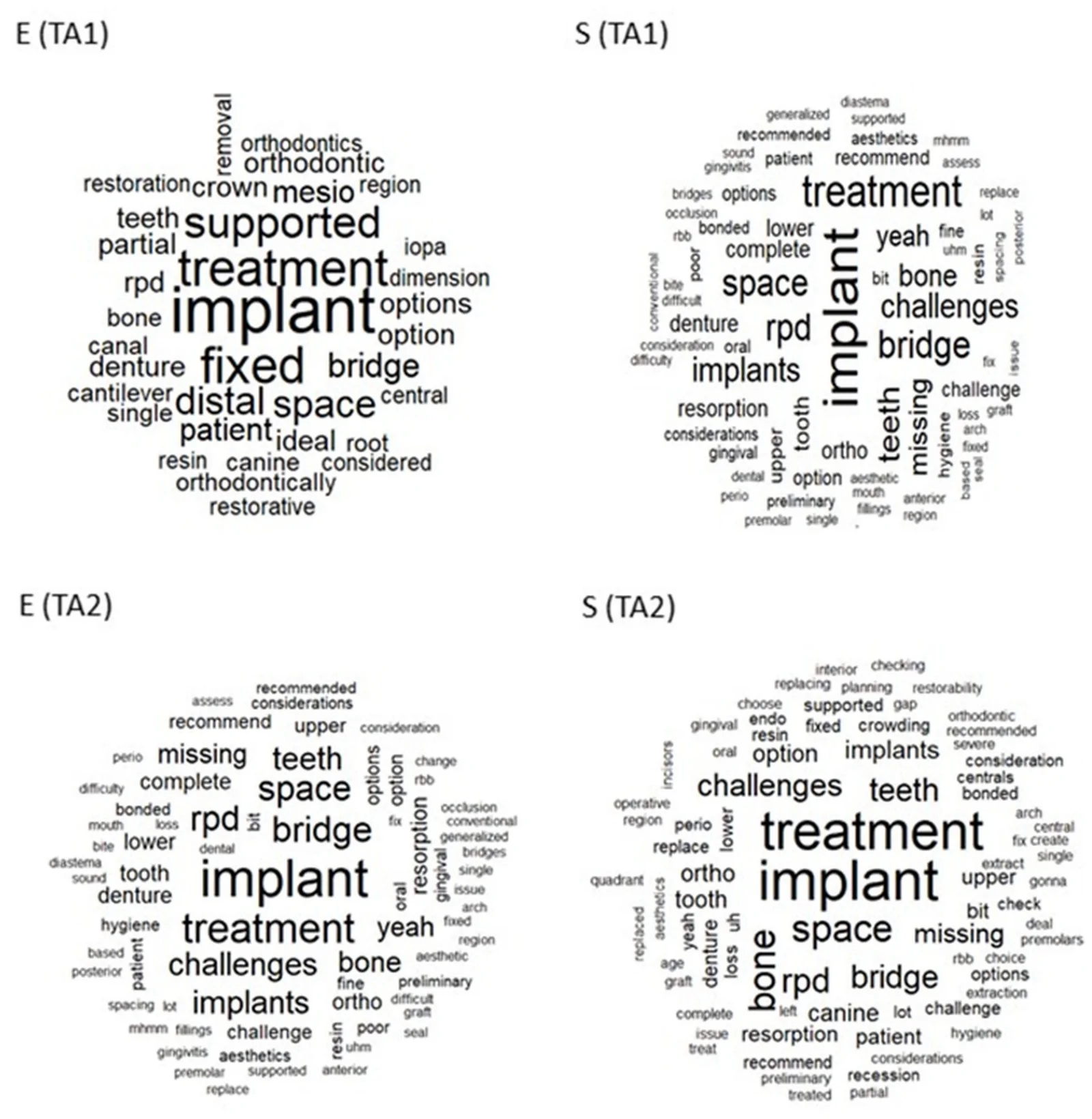
Word cloud of shared terms. TA: Think-aloud, E: Expert, S: Student.

For TA1, the number of shared words was computed to be 65 (Jaccard similarity: 0.19), with “Implant”, “treatment”, “bridge”, “space” and “rpd” being the top five words used. For TA2, the number of shared words was computed to be 90 (Jaccard similarity: 0.23), with “Implant”, “treatment”, “space”, “bridge” and “bone” being the top five words used (Figure 7).

## 4. Discussion

The study explored the use of think-aloud videos as a learning resource to expose students to the thought process of an expert to supplement and support their learning experience.

The content used to prepare the cases was obtained from the clinical cases being treated by the cohort concurrently during the same academic year. Scaffolding and integrating the teaching of clinical reasoning in the undergraduate dental curriculum has been proven efficacious [21]. Individualized and context-based selection of clinical cases to develop appropriate teaching content is proposed to augment the clinical reasoning skills [22]. Conventionally, in clinical teaching, meaningful interactions take place between a clinical instructor and a student in a clinical environment. The student may model their thinking process based on their interaction with the instructor [23]. However, the interaction is limited to the student pertaining to the clinical case in question. The learning moment is not shared with the larger cohort, even though this may be incorporated later in a case-based learning session or interactive reflective session between students [24]. The use of recorded think-aloud videos as a learning resource leads to simultaneous and concurrent sharing of the clinical learning moment with the entire cohort. Each student may use the moment to listen, understand the thought process, agree or disagree thus developing an inherent cognitive process.

Prior experience in thinking aloud is important to create good-quality learning resources. The current teaching instructor who prepared the videos for the current study has previous experience in the effective use of think-aloud videos to supplement clinical reasoning in pre-clinical [19] and clinical learning in prosthodontics [20]. The students were trained in the process of thinking aloud prior to generating their own recordings from clinical cases as recommended [25]. The interactive remote learning experience thus created by the process of making their own recordings, listening to the expert recordings and repeating the generation of recordings encompasses a thorough and prolonged learning experience.

The knowledge test that was conducted by using 10 MCQs supplemented the learning by being aligned with the learning outcomes expected to be covered by the clinical cases. No significant difference was observed between the scores before and after exposure to the think-aloud videos of the expert. This may be explained by the focus of the think-aloud videos on enhancing clinical reasoning in students and may not directly impact the student knowledge considering the short-term impact.

The transcripts were coded into respective themes of pre-treatment considerations, treatment options and challenges. The traditional method of context-free coding was deemed inadequate for the study protocol as identification of decision-making themes which are inherently a part of clinical reasoning may be lost [26]. Alternatively, an a priori coding scheme is an alternative method where the coding is performed prior to the think-aloud sessions to quantify the verbal data into appropriate thematic areas. However, this approach is more commonly employed in a conventional think-aloud interview followed by protocol analysis [27,28].

The Cohen’s kappa values between the students and the expert increased after the students were exposed to the expert think-aloud recordings, but the changes were not statistically significant. The exploratory study may further be enhanced by employing longer term and continuous exposure to think-aloud recordings which may in turn result in a more profound impact on the students. Deliberate practice and maintenance have been previously shown to enhance educational outcomes in dental students [29]. The percent agreement between the students and the expert consistently increased after exposure to the think-aloud recordings in the domains of pre-treatment considerations and challenges. Both these domains are highly dependent on clinical experience and may require years of experience, or exposure to a wide variety of clinical cases before the students may attain an acceptable level of competence [30,31]. The increase in percent agreement was consistent across all cases with minor variation for case 1 which is inherently expected and reflects the individualized cognitive patterns as previously observed and documented [32]. It was interesting to note the decrease in percent agreement pertaining to treatment options before and after the students were exposed to the think-aloud recordings. This may be explained by the level of knowledge for the undergraduate students regarding available treatment options particularly pertaining to the employment of orthodontic tooth movement in the management of missing tooth spaces [33,34]. The word clouds that were generated demonstrated that the expert utilized orthodontic treatment as an option more prominently than the students. This signifies the application of elements derived from think-aloud analysis to enhance the instructional aspect of a course. The existing educational materials utilized for the instructional component of the course may be revised to underscore the significance of space evaluation and prosthodontic therapy in addressing edentulous areas. Moreover, complete agreement in all domains and circumstances following a brief exposure to think-aloud recordings from a single expert may not reflect a realistic outcome. In the future, exposure to think-aloud recordings from various clinical professionals may assist the cognitive growth of undergraduate learners, considering the distinctiveness and creativity of each student [35]. Therefore, the main objective of exposure to the recordings is to enhance cognitive processes, including individuality, rather than to enforce uniformity within a rigid framework. The increased lexical overlap at TA2 may reflect exposure to the expert’s terminology and should not be interpreted as evidence of improved clinical reasoning.

There was a change in congruent themes between the students and expert after exposure to the expert recordings. Even though the students were able to identify challenges and consider alternative treatment options initially, after listening to the expert recordings, a change toward clearer determination of appropriate treatment options and a comparative approach to feasible options were observed in congruence with the expert. The use of think-aloud recordings may facilitate elaborative discussion and help theoretically decrease the cognitive load leading to theme congruence and enhanced learning [36]. Elaborative discussion facilitated by explaining one’s own thoughts during decision making has been shown to enhance learning compared with individual self-explanation [37]. The spaced activities of recording one’s own clinical reasoning, listening to expert recordings, and subsequently generating further recordings may be conceptually related to distributed practice, which has been reported to be more effective than massed learning activities [36,38,39].

The use of expert recordings to support clinical reasoning in novice learners may also be conceptually related to the use of worked examples [36,40]. Worked examples may be particularly beneficial for learners with limited prior knowledge, although their effectiveness depends on the learner’s level of expertise [40]. Excessive information presented during a single teaching session may increase cognitive load and potentially interfere with learning [41]. Therefore, the use of think-aloud recordings may share characteristics with worked examples by allowing novice learners to observe an expert’s reasoning process; however, cognitive load was not measured in the present study, and this interpretation remains theoretical [36,40].

This study has several limitations. The instructional material was developed by a single educator which may limit diversity of perspectives. The university’s instructional framework designates a single course teacher to lead lecture delivery and serve as the primary instructor for the course. The instructional material for the course is provided by this singular instructor. The information for video generation and the questions were evaluated by reviewers to guarantee quality and evidence-based presentation. The content generation approach is customized for this particular university. In instructional styles including numerous educators, an alternative approach must be adopted. The think-aloud recordings may involve discussions of the case among instructors, and standardization meetings may be held.

Transcript length was not normalized for the lexical comparison; therefore, shared-word counts, word-cloud patterns, and Jaccard similarity were interpreted as descriptive measures of lexical overlap rather than as measures of improvement in clinical reasoning.

As individual student engagement with the recordings was not monitored, the extent and frequency of exposure to the intervention cannot be established. The use of different clinical cases at TA1 and TA2 may have introduced case-related variation despite standardization of the learning outcome, vetting process, and case complexity.

The exploratory study was conducted on a very small sample size and a low participation rate (6.7%). However, exploring novel ideas with preliminary studies followed by confirmatory studies is a cautious approach that avoids unnecessarily subjecting students to experimental conditions.

The think-aloud capabilities of instructors and students may pose challenges to the generalizability of this pedagogical approach [42]. Individual, cultural, and environmental limitations in employing a think-aloud approach may necessitate personalized training, guarantees of safety and anonymity, and modifications to institutional protocols. Nonetheless, overcoming initial barriers may foster an environment of trust and cognitive development, rendering the effort worthwhile. This is a core concept in relational pedagogy that emphasizes the interaction between instructor and student, contributing to a secure and joyful learning environment.

Formulating thoughts necessitates prolonged and intensive interaction between students and teachers, well above the brief involvement shown in the current study. This exploratory project establishes the groundwork for a deeper connection between students and educators in a dental education context. The strategy may be particularly suited to postgraduate education with limited student numbers and in-depth discussions of complex treatment plans.

Moreover, think-aloud recordings may offer a distinctive opportunity for student evaluation about the contemporary issues encountered by conventional assessment methods due to the use of AI [43]. The recordings may provide the teacher with a distinctive perspective on student comprehension, facilitating precise evaluation and enhancing the reliability of the assessment.

## 5. Conclusions

This exploratory investigation identified different patterns of student–expert agreement. Agreement increased for pre-treatment considerations, remained relatively stable for anticipated challenges, and decreased for treatment options. Longitudinal studies with a larger sample size are recommended to study these trends. Think-aloud recordings warrant further investigation as a potential educational resource to enhance clinical reasoning.

## Figures and Tables

**Table 1:** Kappa values for student agreement with the expert.

Student	Agreement with Expert (Kappa Scores)	
	TA1	TA2
1	0.15	0.27
2	0.15	0.39
3	0.36	0.36
4	0.29	0.42
5	0.26	0.30
6	0.29	0.24
7	0.13	0.36
8	0.42	0.30
9	0.21	0.24
10	0.32	0.36

**Table 2:** Themes from verbal transcripts (TA1). * Verbal transcripts are italicized.

Themes (Expert)	Expert Verbal Transcript * (Example)	Themes (Students)	Student Verbal Transcript * (Example)
Identification of challenges	*“There is a buccal bone defect which is visible. So, this would need bone augmentation before the implant treatment mostly”*	Identification of challenges	*“The challenges, I think there is bone resorption in the maxilla, so it might be a bit hard to have a good, like a seal, an oral seal.”*
Consideration of alternative treatment options	*“Now, to replace the second premolar, the most ideal choice would be to do an implant supported single crown or a fixed-fixed bridge or a RPD. In that order. A RBB or a cantilever might not be a good option in these cases, especially due to the widened mesiodistal dimension. ”*	Consideration of alternative treatment options	*“I’d go for a removable partial denture, and then an implant, and then my 3rd option would be no treatment.”*
Clear determination of most suitable options	*“The best option would be to do a single implant supported crown for each of those teeth, and the second-best option would be to do a resin bonded bridge.”*	Suggestive approach to treatment options	*“And then for the, for the challenges. I personally don’t see any challenges in this case.”*
Comparison of alternative treatment options	*“In this case, because it includes the canine for the fixed-fixed, and it includes the central for cantilever. An implant or a resin bonded bridge would be the most suitable options.”*	No comparative approach of alternative treatment options	*“I would recommend the implants for this patient only.”*
Firm decisions on initial steps and key areas	*“So, an implant or a resin bonded bridge would be the most suitable options.”*	Consideration of patient preference	*“Based on the preference of the patient or based on his age. I’d go for a removable partial denture, and then an implant, and then my 3rd option would be no treatment.”*
Identification of concerns	*“The 1st concern when I see the picture, for this case is the mesiodistal space. The mesiodistal space seems to be a bit too wide to replace a central lateral incisor, though, and I can see gingivitis on the lower teeth as well.”*	Personal preference	*“But I think I would go for an implant personally. I think he has enough space for an implant.”*
		Considering cost of treatment	*“I don’t see any challenges here. Maybe the cost of the implants.”*

**Table 3:** Themes from verbal transcripts (TA2). * Verbal transcripts are italicized.

Themes (Expert)	Expert Verbal Transcript * (Example)	Themes (Students)	Student Verbal Transcript * (Example)
Identification of challenges	*“Now the additional challenge in this case is that there’s only mandibular anterior is present and the maxillary has only two teeth, and this can lead to combination syndrome once the maxillary teeth are extracted as well.”*	Identification of challenges	*“Okay, first of all, the first challenge is the amount of bone resorption. Replacing the teeth with either complete denture or RPD is going to be difficult because there is not enough bone. Other than that, we have to take a look into the endo treated teeth, I think the lower incisor needs re-treatment and the upper one as well.”*
Consideration of alternative treatment options	*“So, a preliminary treatment consideration would be to do orthodontic assessment and treatment for sure. The second option would be to do resin bonded bridges while using the lateral incisors as abutments.”*	Consideration of alternative treatment options	*“And after we do so, going for implants would be the best option, since it’s in the aesthetic area, and it’s like one of the most important teeth to patients. Usually the central incisors. We could also go for a bridge. A conventional bridge or a resin bonded bridge.”*
Clear determination of most suitable options	*“So, to summarize, the first option is an implant supported fixed processes. The second option is an implant supported removal processes, and the third option is to do interim acrylic removal partial denture prosthesis and transfer the patient slowly to a complete denture, which can be changed into an implant supported processes later.”*	Clear determination of most suitable options	*“The 1st step for us to do is going to be Ortho treatment because she has moderate crowding. So we need more space to replace that missing canine.”*
Comparison of alternative treatment options	*“So, in this case, I would, actually manage the occlusion in such a way that I can go ahead and do a single tooth implant, first of all. Second option, I would still consider an resin bonded bridge and a cantilever bridge, which usually which uses only the premolar and the study as a as an abutment, considering that I can manage the exclusive contacts very, very well.”*	Comparison of alternative treatment options	*“Okay for the treatment considerations. We, of course, have to consider the aesthetic. And then again, for the challenges in the case. I think it’s the scallop on the gingiva. It’s very. I think it’s between high to moderate. So and the placing an implant is gonna it’s gonna make it less aesthetically acceptable. But again, the most suitable is going to be an implant or a resin bonded bridge or an Rpd. But again, aesthetic is one of the biggest considerations.”*
Identification of concerns	*“So, one of the concerns that I see is the presence that there’s only unilateral presence of abutments here.”*	Firm decisions on initial steps and key areas	*“I think I would recommend implants only. Because the space between the teeth is very, very tiny. So, I think, implant is the best choice for this.”*
Identification of case complexity	*“So now there are only unilateral abutment present. So, to use these abutments for an overdenture it seems to be counterintuitive, because I don’t have bilaterally strong teeth present to support an overdenture. And, for the mandibular, posteriorly, the canine seems to be very close, to the ridge, signifying a lot of, bone resorption. So, I don’t expect much of support for my removable partial denture there.”*		

## Data Availability

The student and expert transcripts for both phases and the knowledge test questions and results are available in (DOI 10.5281/zenodo.21850449).
